# Unmet need for interprofessional education in paediatric cancer: a scoping review

**DOI:** 10.1007/s00520-019-04856-4

**Published:** 2019-05-24

**Authors:** Martha Krogh Topperzer, Marianne Hoffmann, Louise Ingerslev Roug, Hanne Bækgaard Larsen, Birgitte Lausen, Kjeld Schmiegelow, Jette Led Sørensen

**Affiliations:** 1 Paediatric Oncology Research Laboratory, Department of Paediatrics and Adolescent Medicine, Rigshospitalet, University of Copenhagen, Blegdamsvej 9, 2100 Copenhagen, Denmark; 2 Department of Paediatrics and Adolescent Medicine, Rigshospitalet, University of Copenhagen, Copenhagen, Denmark; 3 Juliane Marie Centre for Children, Women and Reproduction, Rigshospitalet, University of Copenhagen, Copenhagen, Denmark

**Keywords:** Interprofessional education, Evaluation, Curriculum, Paediatric oncology, Cancer

## Abstract

**Purpose:**

Despite improved treatment and care, children and adolescents diagnosed with cancer continue to die, while many of those cured are burdened by treatment-related sequelae. The best clinical management of children and adolescents with cancer depends on healthcare professionals with various skills and expertise. Complex treatment, care and rehabilitation require collaboration between healthcare professionals. The purpose of this scoping review is to identify and evaluate existing interprofessional education in paediatric cancer.

**Methods:**

We utilised the scoping review methodology and searched PubMed, Scopus and Education Resources Information Center. Inclusion criteria were postgraduate studies targeting more than one profession and evaluation of the educational intervention. We applied Kirkpatrick’s modified interprofessional education outcomes model to systematise outcomes.

**Results:**

Of 418 references, nine studies fulfilled the inclusion criteria. The design, strategy and content of all the studies were heterogeneous. None of the interprofessional educations systematically evaluated knowledge, skills, attitudes or the effects on patient outcomes or quality of care.

**Conclusion:**

There is a lack of well-structured, interprofessional education in paediatric cancer that has undergone evaluation. Paediatric cancer may benefit from systematic education and evaluation frameworks since interprofessional education could potentially strengthen the treatment, care and rehabilitation for children and adolescents with cancer.

**Electronic supplementary material:**

The online version of this article (10.1007/s00520-019-04856-4) contains supplementary material, which is available to authorized users.

## Introduction

Despite improved treatment and care, children and adolescents diagnosed with cancer in the Western world continue to die, while many of those cured are burdened by treatment-related sequelae [1, 2]. The best clinical management of children and adolescents with cancer depends on healthcare professionals with various skills and expertise [3] as the treatment, care and rehabilitation of children and adolescents with cancer is so complex that it surpasses the responsibilities and abilities of one single profession. To provide the best treatment and care, healthcare professionals are thus required to collaborate [4, 5] and interprofessional teams appear to be a vital component of the quality of care for children and adolescents with cancer and their families [6, 7]; however, the evidence supporting this remains limited.

In the process of designing an interprofessional education in paediatric cancer at Rigshospitalet, the largest paediatric cancer department in Copenhagen, Denmark, this research group found it relevant to explore if any interprofessional education in paediatric cancer existed. A steering group was established comprising oncological consultants (MHH, BL), the professor (KS), the head nurse of the Children and Adolescents Unit (MMA), the head nurse of the paediatric cancer department (PR), the leader of psychosocial research in Laboratory of Paediatric Oncology (HBL) and head of education and associate professor (JLS and PhD student (MKTOP)).

Interprofessional education should be strategically planned based on a curriculum to continuously ensure and strengthen high-quality care for children and adolescents with cancer and their families. In medical education, various frameworks exist [8–10], such as the six-step approach to curriculum development [11].

A curriculum can be defined as “a planned educational experience” [11] that includes short- and long-term learning experiences. The curriculum comprises problem identification, needs assessment, aims and objectives, educational strategies, implementation, assessment and evaluation and feedback [11].

Interprofessional education can be defined as “occasions when two or more professionals learn with, from and about each other to improve collaboration and the quality of care” [12]. A systematic review of the effects of interprofessional education identified empirical research that supports the underlying assumption that interprofessional education enhances the delivery of safe, high-quality care for patients [13]. Further, that learners react positively to interprofessional education by improving collaborative attitudes and perceptions, and report improvements in both knowledge and skills on a variety of outcomes [13].

This assumes that an education intervention improves how healthcare professionals work together, which in turn may lead to improved patient outcomes [13]. Interprofessional education has been established and in some settings shown to have a positive impact on the knowledge, attitudes and behaviours of healthcare professionals [14]. To derive the most benefit from educational interventions, medical education can be viewed as a health technology applying evidence-based practice and evaluation for clinical practice [15]. However, interprofessional outcomes are not easily monitored and research addressing interprofessional education is inherently complex [13, 16].

Curriculum outcomes typically cover cognitive (knowledge), psychomotor (skills) and affective (attitude) objectives, as defined by Bloom’s taxonomy [11]. A robust evaluation design is essential to report changes in the knowledge, skills and attitudes of healthcare professionals [14, 17, 18]. According to Kirkpatrick’s outcome evaluation model, which dates from the 1950s [10, 19], learning takes place when a change is registered in knowledge, skills or attitudes. The model pragmatically assists in framing potential areas and purposes of evaluation. Kirkpatrick’s model has been widely applied in the assessment of interprofessional education [20]. Barr and colleagues extended the model to capture more detailed outcomes relevant to interprofessional education and also incorporated a level of benefits to patients as shown in Table 1 [14, 20].

**Table 1:** Classification of Kirkpatrick’s interprofessional education outcomes model modified by Barr et al. 2005

Level	Outcome	Details
Level 1	Reaction	Learner’s views on the learning experience and its interprofessional nature
Level 2a	Modification of attitudes/perceptions	Changes in reciprocal attitudes or perceptions between participants groups. Changes in perception or attitude toward the value and/or use of team approaches to caring for a specific client group
Level 2b	Acquisition of knowledge/skills	Including knowledge and skills linked to interprofessional collaboration
Level 3	Behavioural change	Identifies individuals’ transfer of interprofessional learning to their practice setting and their changed professional practice
Level 4a	Change to organisational practice	Wider changes in the organisation and delivery of care
Level 4b	Benefits to patients/clients	Improvements in health or well-being of patients/clients

This model describes evaluation of educational programmes and is based on Kirkpatrick [19] and modified to interprofessional education by Barr et al. [20]

Health education research has widely applied scoping reviews [21–25] to identify key concepts in specific research areas, especially complex ones that have not been reviewed earlier [26]. According to Arksey and O’Malley, a scoping review can examine the extent, range and nature of research activity; determine the value of undertaking a full systematic review; and summarise and disseminate research findings but also identify research gaps in the existing literature [26]. The scoping review methodology differentiates from other review methods such as the Preferred Reporting Items for Systematic Reviews and Meta-Analyses (PRISMA) in several ways [27]. Most notably, the research questions for scoping reviews are more broadly defined compared with systematic reviews’ research questions. This leads to the inclusion of all types of methods as opposed to specific methods in systematic reviews [27]. Scoping reviews can contribute to generating hypotheses and chart the data according to key issues rather than synthesizing and aggregating findings as in a systematic review [26, 28].

The purpose of this scoping review is to identify and evaluate existing interprofessional education in paediatric cancer.

## Methods

We applied Arksey and O’Malley’s scoping review stages 1–6 [26]. Table 2 provides an overview of how we applied the scoping review stages in this study.

**Table 2:** Application in this study of scoping review methodology based on Arksey and O’Malley and inspired by Reeves et al. 2017

Review stage based on Arksey and O’Malley [26]	Specifications on how we applied stages in this study
1: Identifying the research question	In the present study, we defined broad inclusion criteria to encompass the wide-ranging aspects of education planning and evaluation in paediatric oncology and to find as many relevant articles as possible; defining keywords such as paediatric, oncology and haematology has implications for the depth and range of included studies.
2: Identifying relevant studies	We searched databases for identification of relevant studies; scoping methodology permits comprehensive searches of e.g. electronic databases, lists of articles, conference papers and grey literature, such as websites.
3: Study selection	We applied broad inclusion and exclusion criteria both before and after the search and subsequently defined which job titles were monoprofessional and determined if an intervention was presented.
4: Charting the data	In this study, we decided which information to register and how to compare the various interventions before sorting the material; key issues relevant to the research question, such as education topics, types of healthcare professionals and evaluation methods were included.
5: Collating, summarising and reporting the results	The purpose of a scoping review is not to present evidence the way a systematic review does but to use the reviewed material to help present an overview; we reported data in relation to two theories relevant to medical education: Kern’s six-step [11] approach to curriculum development, to assess the educational content, and the interprofessional education outcomes model [20], to evaluate the outcomes of the identified articles.
6: Consultation (optional)	Consulting with stakeholders about results can help in identifying additional articles and provide new insights; all authors of this scoping review comprised a steering group that discussed the findings and implications on an ongoing basis.

### Stage 1: Identifying the research question

In the process of designing an interprofessional education in paediatric cancer, a literature search was needed to identify existing education. Our research question was formulated to encompass the broad aspects of education planning and evaluation in paediatric cancer.

Research question:

- What does the literature reveal about interprofessional education in paediatric cancer?

With this broad research question, we wish to examine the extent, range and nature of educational activities in paediatric cancer, specifically explore if and how education programmes are evaluated and determine the nature of the reported outcomes.

### Stage 2: Identifying relevant studies

An information specialist assisted in generating a search strategy based on keywords involving the research question: (Oncology OR Hematology) AND (“Pediatric medicine” OR Pediatrics OR “Adolescents medicine”) AND (Curriculum OR “Education programme” OR “Educational programme” OR “Interprofessional education” OR “Interdisciplinary education” OR Program Development OR Postgraduate).

**Fig. 1:**
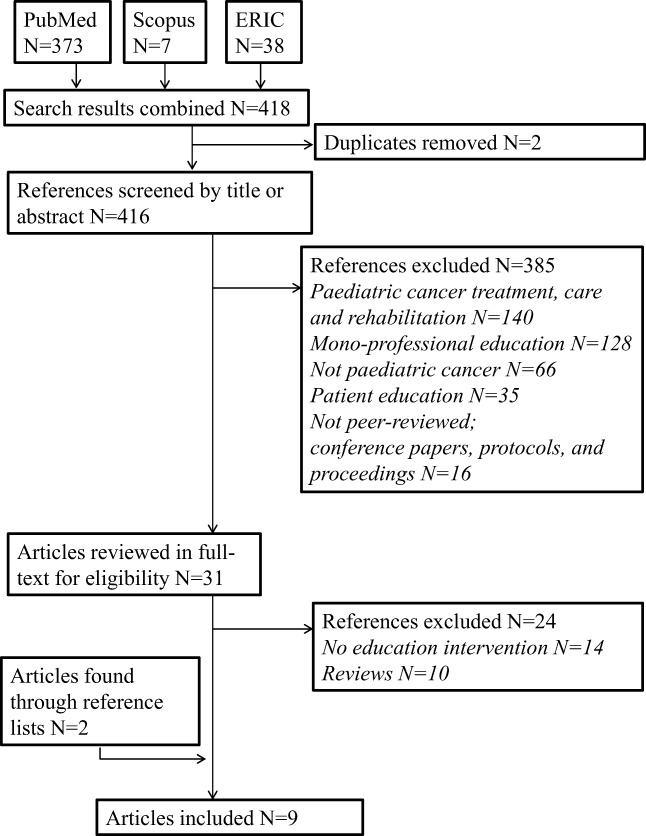
Search strategy and selection of studies

We searched the following databases with educational interventions: PubMed, Scopus and Education Resources Information Center (ERIC). The searches were not limited by date, country of origin or original published language. Figure 1 provides a flowchart of the studies identified and how they were selected.

The scoping review methodology allows for inclusion of grey literature [26], which can be defined as “anything that has not been published in traditional format, or in library parlance, lacks bibliographic control […] this includes […] conference proceedings, conference posters […]” [29].

We searched online for interprofessional education in organisations such as the American Society of Pediatric Hematology/Oncology (ASPHO) [30], the Nordic Society of Paediatric Haematology and Oncology (NOPHO) [31], the Nordic Society of Pediatric Oncology Nurses (NOBOS) [32] and hospital websites, such as MD Anderson Cancer Center [33] and St. Jude Children’s Research Hospital [34].

### Stage 3: Study selection

To answer the research question, we applied the following four inclusion criteria: (1) postgraduate education interventions (2) in the field of paediatric cancer (3) targeting more than one profession and (4) including an evaluation of the education intervention.

The exclusion criteria were monoprofessional education, education in other medical fields and interventions regarding patient treatment, care and rehabilitation or patient education. Figure 1 illustrates the study selection process.

We (MKT, LIR and MH) independently screened titles and abstracts. If the abstract met the inclusion criteria, full-text papers were obtained and assessed individually by two authors (MKT and MH). Full-text articles are included in supplementary 1. In the event of a disagreement, each author provided justification for their decision-making process until consensus was achieved. When the educational or methodological approaches diverged, JLS was consulted, while a second senior consultant (BL) was consulted of divergences involving paediatric cancer.

Articles in languages other than English as elaborated in supplementary 1 were screened based on abstracts written in English and then filtered according to the inclusion and exclusion criteria.

Endnote X8 was used to store all articles, while the web-based programme Rayyan [35] using a semi-automatic process assisted in screening and sorting studies based on abstracts and titles.

### Stage 4: Charting the data

We extracted information from the articles regarding general information such as country of origin. We also extracted specific information about the healthcare professionals involved and the aims, strategies and outcomes of the educational activities.

### Stage 5: Collating, summarising and reporting the results

We reported data from the identified articles in accordance with two theories relevant to medical education: Kern’s six-step approach to curriculum development [11], to assess the curriculum and educational content, and the modified Kirkpatrick outcomes model [20], to evaluate the outcomes.

### Stage 6: Consultation (optional)

The scoping review methodology as formulated by Arksey and O’Malley consists of five steps. However, Arksey and O’Malley suggest that including the opinion of stakeholders such as practitioners and consumers can contribute to applicability of the results. This step is optional to researchers and there is no description on when and how to apply this sixth optional step [36].

We applied the sixth step throughout the iterative process of the scoping review when we presented the findings to the steering group. All authors of this scoping review comprised the steering group that discussed the findings and implications on an ongoing basis.

## Results

The database searches resulted in 418 records, two of which were removed because they were duplicates. Of the 416 records that remained, a further 385 records were excluded. After reading 31 full-text articles, we identified two additional studies from their reference lists. This process led to the final inclusion of nine studies for analysis as shown in Fig. 1. The excluded 24 articles covered reviews (*n* = 10) and 14 articles which did not include an education intervention.

Supplemental information S1 provides an overview of the final nine references’ educational activities, an overview of the final 9 references’ methodological information, overview of the 33 full-text articles, articles in languages other than English and the number of citations in Scopus.

No relevant interprofessional education was identified in the searched grey [29] literature. Included studies’ dates ranged from 1967 to 2017.

MH and MKT consulted with JLS on four occasions to resolve whether full-text articles should be included or excluded.

Results were sub-classified as existing interprofessional education as shown in Table 3 and evaluation of interprofessional education as shown in Table 4.

**Table 3:** Study characteristics of the nine included studies

Citation, country and study design	Healthcare professionals *N*	Educational intervention	Aim of education/intervention	Duration	Theory	Educational strategies	Assessment method	Accreditation of programmes/assessment of individuals
Bouri et al. 2017 Greece Pre-post study comparing intervention group with control group measured by questionnaires	37 nurses, 21 psychologists, 10 paediatricians and 15 other disciplines (social workers, music therapists, and physiotherapists) *N* = 83	Paediatric palliative training of health professionals on attitudes toward death	To advance participant’s knowledge, skills and attitudes in seven domains: pain, communication, ethics, psychosocial and spiritual care, bereavement support, interdisciplinary teamwork and self-awareness and reflective practice	150-h training programme, 8 months, 2 sessions per month	No theory identified	Lectures, video presentation, discussion of case studies, role play exercises, group discussions, self-reflection exercises. Each participant accompanied a patient throughout the course	The Death Attitude profile-revised (Wong, Reker and Gesser 1994)	Formative assessment
Di Giulio et al. 2013 13 European countries Descriptive report, reporting on projects implemented, publication of results and focus group	10 teams of unspecified number of nurses and physicians	Collaboration between physicians and nurses	To collaboratively explore and facilitate professional groups to work together more effectively	42-day seminars held over a 4-year period	Appreciative inquiry	No specific mention of strategies	1: Elaboration of projects for integrated activities between doctors and nurses 2: Number of projects implemented and successfully completed 3: Publication of the results 4: Feedback from participants on their perception of improvement of collaboration	n/a
Dobrasz et al. 2013 USA Retrospective study reviewing medical records	Registered nurses, paramedics, patient care technicians and physicians *N* = 271	Nurse-driven protocols for febrile paediatric oncology patients	To evaluate the impact of an evidence-based practice change to streamline the door-to-drug process of prompt identification of febrile patients and initiation of antibiotic therapy	Study period 2008–2012 No further mention of frequency or length	No theory identified	Staff meetings, between shift rallies, weekly e-mail update, skill stations annual competency assessment, visual tracking board	2758 medical records	n/a
Finley et al. 2008 Jordan Action research using mixed methods; qualitative and quantitative	Two groups: Group I: 4 physicians, 2 nurses, 1 pharmacist. Group II: 14 staff nurses, 14 physicians *N* = 31	Paediatric pain policy and procedures	To execute a capacity building programme to develop, implement and evaluate a paediatric pain management programme	Three 10-day visits over a 2-year period	No theory identified	Educational sessions (no further information)	Chart audit Field observations	n/a
Moody et al. 2013 USA/Israel Pre-post study comparing the intervention group with the control group measured by questionnaires	Nurses, social workers, physicians, nurse practitioners, psychologists and child-life specialists *N* = 48	Mindfulness training for burnout	To decrease burnout in a multidisciplinary group	15 h over 8 weeks: one initial 6-h session; 6 weekly 1-h follow-up sessions; final 3-h wrap up session	Mindfulness training by Kabat-Zinn	Didactic material topics included “cultivating awareness of body sensations, thoughts, and emotions […] exploring individual reactivity to stress, reflecting on meaningful experiences and practice, training in skilful listening, and communication and self-care” Formal meditative practices, daily logs	1) Maslach Burnout Inventory 2) Perceived Stress Scale-14 3) Beck Depression Inventory	n/a
Neyrinck et al. 2015 Indonesia Pre-post study comparing participants knowledge before and after intervention	38 apheresis nurses and 32 physicians *N* = 70	Apheresis training	To hold a certification course for apheresis nurses/operators based on a training programme	10 modules	No theory identified	Presentations, slides and training the trainer. No further didactics mentioned	Multiple choice test, yes-no and open questions	Non-validated certification
Sands et al. 2008 USA Pre-post study comparing baseline with post intervention measures based on questionnaire and focus groups	6 physicians, 12 nurses and 1 psychosocial member *N* = 19	Interprofessional training to promote empathy, build teams and prevent burnout	To execute a feasibility and effectiveness study of narrative training	Weekly seminars for 6 weeks	Narrative medicine	Participants wrote and read aloud Facilitated discussion	Baseline and post intervention assessment using Interpersonel Reactivity Index and Stressor Scale for Pediatric Oncology Nurses Focus group of 14 participants in 1 h	No accreditation
Treadwell et al. 2002 UK Pre-post study comparing baseline with post intervention measures based on structured interviews	T1: 36 children and 68 staff (36 physicians, 29 nurses and 3 psychosocial staff) *N* = 104 T2: 49 children and 82 staff (41 physicians, 35 nurses and 6 psychosocial staff) *N* = 131	Quality improvement of paediatric pain assessment	To evaluate the impact of a quality improvement approach to implementing developmentally appropriate pain assessment guidelines	Not defined	No theory identified	Staff education included “didactics, discussion and role plays” no mention of specific amount of time	Patient outcome questionnaire developed by American Pain Society for quality improvement of acute and cancer pain modified according to age groups	No accreditation
Zernikow et al. 2008 Germany Longitudinal national quality improvement study based on questionnaire and semi-structured interviews	76 heads of departments/supervising physicians, 46 ward physicians, 63 head nurses, 44 psychologists and social workers *N* = 229 363 children and 46 parents were also interviewed	Quality improvement of paediatric pain control	To evaluate a quality improvement study to improve paediatric oncology pain control in Germany	Not defined	No theory identified	Two regularly scheduled formal education sessions on paediatric pain	Questionnaire (not validated) on knowledge on pain Standard pain documentation sheet Semi-structured interviews	n/a

**Table 4:** Application of Kirkpatrick’s modified interprofessional education outcomes model by Barr et al. 2005

Study	Educational intervention	Kirkpatrick levels	Main outcomes
Bouri et al. 2017	Paediatric palliative training on attitudes toward death of health professionals	Level 2a Modification of attitudes	Higher scores in the intervention group than control group in all measurements
Di Giulio et al. 2013	Collaboration between physicians and nurses	Level 1 Reaction	Impressions of being involved
		Level 2a Modification of attitudes/perception	Participants “felt that their attitude to[ward] collaboration had improved”
		Level 2b Acquisition of knowledge/skills	Five teams published in non-peer-reviewed publications and presented outputs at conferences
Dobrasz et al. 2013	Nurse-driven protocols for febrile paediatric oncology patients	Level 3 Behaviour change	Increased compliance with protocol Faster response to administration of drugs
		Level 4b Benefits to patients/clients	Decreased length of hospital stays Reduced systemic infection and mortality
Finley et al. 2008	Paediatric pain policy and procedures	Level 2a Modification of attitudes/perception	After implementation of programme, physicians and nurses administered opioids, and continuous opioid infusions were used for various types of pain
		Level 2b Acquisition of knowledge/skills	Increased assessment of children’s pain
		Level 3 Behaviour change	Daily informal teaching and consultation
Moody et al. 2013	Mindfulness training for burnout	Level 2a Modification of attitudes/perception	Assessment of emotional exhaustion, depersonalisation and personal accomplishment
		Level 3 Behavioural change	Mindfulness of one’s actions and awareness of the effects of working with this patient population Greater focus and efficacy at work
Neyrinck et al. 2015	Apheresis training	Level 2b Acquisition of knowledge/skills	Nurses and physicians increased their knowledge significantly
Sands et al. 2008	Interprofessional training to promote empathy, build teams and prevent burnout	Level 2a Modification of attitudes/perceptions	Increased ability in the “perspective training” domain Improvement in the “empathic concern” domain Perceived stress levels increased
Treadwell et al. 2002	Quality improvement of paediatric pain assessment	Level 2a Modification of attitudes/perceptions	Significant increase in staff satisfaction
		Level 3 Behavioural change	Significant increase in pain assessment Increased compliance with pain assessment documentation guidelines
		Level 4b Benefits to patients/clients	Patients and caregivers reported significant increase in the staff’s use of pain measures Increased staff responsiveness and greater use of adjunctive pain management strategies
Zernikow et al. 2008	Quality improvement of paediatric pain control	Level 2b Acquisition of knowledge/skills	Increased application of pain scale Significant decrease in painful modes of analgesic administration Increased use of pure u-opioids agonist Knowledge improvement of neuropathic pain treatment
		Level 4b Benefits to patients/clients	Significant reduction of daily pain intensity rated by patients and parents Significant decrease in severe pain frequency reported by patients and parents

### Existing interprofessional education

The number of participants in each study varied from 19 [37] to 229 [38]. The healthcare professionals represented in the studies were predominantly nurses, physicians and psychosocial staff [37–39]. These groups of interprofessional healthcare professionals were supplemented in one study by a child-life specialist [40], a pharmacist [41] and a music therapist [42]. Two studies only targeted physicians and nurses [43, 44], and one study supplemented these two groups of healthcare professionals with paramedics and patient care technicians [45].

The topics that the interprofessional educations covered included pain management and assessment [38, 39, 41], team training to prevent burnout [37, 40], collaboration of healthcare professionals [43], training on the attitudes of healthcare professionals toward death [42], apheresis training [44] and improving initiation of antibiotics for febrile patients [45].

Learning activities and educational strategies covered in the included studies are seminars [43], educational sessions [38, 41], lectures [42], staff meetings [45], slide presentations [44] and activities such as role play [39], reflections [37] and formal meditations [40].

### Evaluation of interprofessional education

Five studies were pre-post intervention studies that compared baseline measurements with outcomes following an intervention [37, 39, 40, 42, 44]. Three studies had control groups [38, 40, 42], one of which randomised participants to either the control or intervention group [40]. Data collected included questionnaires on knowledge [38, 44] and attitudes [38, 42] and information gathered in focus groups [37, 41] and structured interviews [39]. One study collected data from medical records [45], and one training programme offered certification of the skills acquired; however, there was no validation of the certification [44].

None of the identified articles applied a medical education or curriculum model, such as the six-step approach, to curriculum development [11], or Harden’s “ten questions to ask when planning a course or curriculum” [46].

None of the nine studies applied systematic evaluation theory to participant assessments in terms of knowledge, skills, attitudes or the effects on patient outcomes, such as quality of care. However, six studies reported statistically significant findings concerning knowledge [38, 44], behaviour change [39] and attitudes [37, 40, 42].

We applied Kirkpatrick’s [19] modified model [20] to systematise outcomes across the interventions identified for close analysis as shown in Table 4.

One study reported on the reaction of participants to being part of the intervention [43] (level 1) [20]. Three studies reported on acquisition of knowledge [38, 41, 44] and four studies [37, 40, 42, 43] evaluated the modification of attitudes among healthcare professionals [41] (level 2) [20].

Four studies measured behaviour change outcomes (level 3) [20], including increased compliance to guidelines [38, 39, 45] and increased self-awareness [40].

Three studies [38, 39, 45] reported on level 4b [20] that cover improvements in the health of patients.

## Discussion

There is a lack of well-structured, interprofessional education in paediatric cancer that has undergone evaluation. We found few studies that assessed the needs of learners or defined the healthcare needs of the patients. Most studies planned the educational activities according to available standards, competency frameworks and organisational demands.

In the definition of interprofessional education, “occasions when two or more professionals learn with, from and about each other to improve collaboration and the quality of care” [12], the focus is on improving collaboration and the quality of care. We only identified one study [37] with an explicit interprofessional aim. However, there are many definitions of interprofessional collaboration and interprofessional practice which are also sometimes referred to as team work [47]. We adhere to the contingency approach of interprofessional practice as formulated by Reeves et al. that the “design of the team need to be matched to its clinical purpose(s) in order to serve the local needs of patients” [48]. This implies that interprofessional practice depends on two aspects, the clinical purpose and the patients’ needs, and that the choice of which healthcare professionals should collaborate depends on these two aspects.

In designing interprofessional education, focus should be on improving collaboration and heightening the quality of care, relating to i.e. Kirkpatrick’s outcome level 3, which “measures the transfer of interprofessional skills and learning to workplace” [13]. This could be support for behaviour change in the department or willingness of healthcare professionals to apply new knowledge and skills about collaborative work to their practice style.

In medical education, it is fundamental to link curricula to healthcare needs and define aims [11]. Meeting healthcare needs requires an interprofessional approach in many specialties, including paediatric cancer. We can potentially ensure and strengthen treatment and care for children and adolescents with cancer and their families by linking interprofessional education to the healthcare needs of the patients because the best clinical management of children and adolescents with cancer depends on healthcare professionals with various skills and expertise.

Educational strategies were superficially described across studies, and none compared the various effects of educational methods or teaching strategies in the interventions. A transparent presentation of educational methods can inspire other healthcare professionals to develop curricula and evaluate their education programmes [11, 49, 50]. Furthermore, application of a medical education framework to structure the educational intervention would allow hospital management and department managers to hold medical educators accountable [11].

The identified interventions did not follow any specific evaluation framework, making it difficult to compare them in this scoping review. Incorporating an interprofessional evaluation framework in interventions can serve to aid systematic evaluation of the usefulness of education programmes [49]. Even though Kirkpatrick and Barr et al. have been subject to criticism due to the apparent simplicity of the outcomes models [51–54], both models are helpful in the process of planning the evaluation of medical education.

## Limitations of the review

The primary limitation of this scoping review is the low number of included studies making the generalisability of the results difficult. The heterogeneity of the findings challenges the interpretation of the results extracted. To counteract this, we presented our results transparently to increase credibility.

In the nine articles reviewed, self-reported measures were used in evaluating outcomes related to healthcare professional knowledge, skills and attitudes. An inherent weakness in self-reported outcome measurement is that individuals often over- or underestimate their knowledge, skills and behaviours [55, 56]. In this scoping review, three studies reported on acquisition of skills [39, 41, 45]; however, only two studies documented this [39, 45]. Instead, surrogate outcomes such as 24-h chart audits [41] or tests of knowledge of what to do (skill) in case of machine breakdown [44] were used to indicate that an increase in the knowledge of the healthcare professionals was associated with behaviour change.

According to Arksey and O’Malley, the purpose of a scoping review is to aid in determining the value of undertaking a full systematic review [26]. We suggest that the application of a systematic review methodology, such as PRISMA Statement [28], would not currently be feasible due to the heterogeneity and limited number of relevant studies.

Even though the scoping review methodology allows for inclusion of grey literature [29], we did not systematically include it in the findings. It is possible, however, that organisations, such as NOPHO, NOBOS and ASPHO, and hospitals, such as the MD Anderson Cancer Center or St. Jude Children’s Research Hospital, have developed and implemented interprofessional education without publishing or posting online.

## Conclusion

In conclusion, medical education should be viewed similar to any other health technology, which is why evidence-based practice and evaluation for clinical practice in paediatric cancer is necessary to derive the most benefit from educational interventions [15]. This scoping review illustrates the lack of interprofessional education in paediatric cancer.

## Perspectives

Based on the education theory and literature, we recommend that future interprofessional educations apply a medical education framework [11, 46] in designing interventions; select aims and objectives based on a needs assessment [11]; define outcomes before designing the intervention, with patient outcomes included when possible [57]; select topics relevant for an interprofessional education intervention, though some interventions are more relevant for monoprofessional education [58]; and, finally, use of a systematic approach to the evaluation [19, 20] with the allocation of relevant resources [11].

## Electronic supplementary material

The following supplementary material is available for this article: Supplemental 1 (search, full-text articles, languages other than English, Scopus citations and an overview of the nine included studies’ methods and educational activities).

ESM 1 (PDF 171 kb)

## Abbreviations

ASPHO: The American Society of Pediatric Hematology/Oncology
ERIC: Education Resources Information Center
NOBOS: Nordic Society of Pediatric Oncology Nurses
NOPHO: Nordic Society of Paediatric Haematology and Oncology
PRISMA: Preferred Reporting Items for Systematic Reviews and Meta-Analyses
