# Supplementary material for: Unmet need for interprofessional education in paediatric cancer: a scoping review
Source: Support Care Cancer. 2019 May 24;27(10):3627–37. doi: 10.1007/s00520-019-04856-4 (PMC6726701; doi:10.1007/s00520-019-04856-4)
Supplement: Supplementary file 1 — (PDF 171 kb) [file 520_2019_4856_MOESM1_ESM.pdf]

Supplemental material 1, Overview of final nine references educational activities					
Citation	Aim of education/ duration / theory	Participants	Learning methods	Evaluation method	Accreditation / Assessment - relevance?
Bouri et al. 2017	Explore impact of a paeditric palliative care training programme on attitudes, 150 hour training programme, 8 months, 2 sessions per month, no theory identified	37 nurses, 21 psychologists, 10 pediatricians and 15 other disciplines (social workers, music therapists, physiotherapists)	Lectures, video presentation, discussion of case studies, role play exercises, group discussions, self reflection exercises. Accompany a patient	The Death Attitude profile- revised (Wong, Reker and Gesser 1994)	Accreditation not mentioned formative assessment
Di Giulio et al. 2013	Three year seminar; collaborative approach to explore and facilitate professional groups to work together more effectively . Theory used Appreciative enquiry	10 teams of unspecified number of nurses and physicians	4 seminars	1;Elaboration of projects of integrated activities between doctors and nurses, 2; number of projetcs implemented and succesfully completed, 3; publication of the results, 4; feedback from participants on their perception of improvement of collaboration	n/a
Finley et al. 2008	Action research programme drawing on multiple theories; knowledge translation, Promotin Action on Research implementation in health services (PARIHS), Dissusion of Innovation, PDSA	Four physicians, two nurses, one pharmasists , one "lead" researcher (visit one), additional staff; 14 nurses and 14 physicians	not described	assessment of childrens pain (charted), use of cont opiods,	n/a

Moody et al. 2013	15 hours over 8 weeks mindfullness education via a structured, skills-training course	nurses, social workers, physicians, nurse practioners, psychologists, child-life specialists	8 weeks of structured, skills-training course.one initial 6 hour session, 6 weekly 1-hour follow-up sessions; and a final 3-hour wrap-up session.	Diadactic material topics included "cultivating awareness of body sensations, thoughts, and emotions […]exploring individual reactivity to stress, reflecting on meaningful experiences and practice, training in skillfull listening, and communication and self-care". Formal meditative practices, daily logs.	n/a
Neyrinck et al. 2015	Certification course for apheresis nurses/operators based on a training programme	38 apheresis nurses and 32 physicians	10 modules, presentation, slides and train the trainer no further didactics mentioned	Multiple choice test, yes-no, and open questions	Certification but no accreditation
Sands et al. 2008	Feasibility and effectiveness study of narrative training	6 physicians, 12 nurses, 1 psychosocial member	Weekely seminars for 6 weeks; participants wrote and read aloud facilitated discussion	Baseline and post intervention assessment (interpersonel reactivity index and stressor scale for paediatric oncology nurses), focus group	No accreditation
Treadwell et al. 2002	To evaluate impact of a quality improvement approach to iplementing developmentaly appropriate pain assessment guidelines	T1: 36 children and 68 staff (36 physicians, 29 nurses, and 3 psychosocial staff) , T2: 49 children and 82 staff (41 physicians, 35 nurses, six psychosocial staff)	Staff education included "didactics, discussion and role plays" no mention of specific amount of time	Structured interviews adapted from the patient outcome questionnaire developed by American Pain society	No accreditation
Zernikow et al.2008	Evaluate a Quality improvement study with the aim to improve paediatric oncology pain control in Germany	76 heads of departments/supervising physicians, 46 ward physicians, 63 head nurses, 44 psychologists and social workers	2 regularly scheduled formal education sessions on paediatric pain	Survey on knowledge on pain (validated?), document pain therapies using standars pain documentation sheet, semi-structured interviews	n/a

Supplemental material 2, Overview of final nine references methodological information				
Citation	Study design	Data collected	Sample size	Outcome
Bouri et al. 2017	Pre/post intervention	Survey (validated)	n= 83	Increased scores at T2 of Neutral acceptance
Di Giulio et al. 2013	Multicenter study - appreciative inquiry	Projects implemented, publication of results, participants satisfaction	10 teams	1;Elaboration of projects of integrated activities between doctors and nurses, 2; number of projects implemented and successfully completed, 3; publication of the results, 4; feedback from participants on their perception of improvement of collaboration
Finley et al. 2008	Qualitative	Field observation, two focus group, unstructured interviews, individual intervies , audits of patient charts and pharmacy records	n=33	Policies and protocols developed - no elaboration on the education programme
Moody et al	Pre/post intervention	Survey (validated)	n=48	Primat outcome measured burnout by Maslach Burnout inventory. Secondary outcome included perceived stress and depression. Qualitative analyses of journals was used to capture subjective changes as experienced by partipants
Neyrinck et al. 2015	Pre/post intervention	Survey (not validated)	n=70	Certification of nurses and physicians
Sands et al. 2008	pre/post intervention and qualitative (Mixed Methods)	surveys (validated) and focus group	n=19	Qualitative outcomes<br>Increased ability of Perspective taking<br>Trend toward significance of improvement in Empathetic concern<br>No significant changes in Fantasy or Personal distress

Treadwell et al. 2002	Pre/post intervention	Structured interviews adapted from the patient outcome questionnaire developed by American Pain society (validated)	n=T1:104, T2; 131	Patient and staff satisfaction with pain assessment and management and chart audit of compliance with pain assessment documentation
Zernikow et al.2008	Longitudinal nation-wide, multi-centre study	Survey on knowledge and attitudes (validated?), pain intensity ratings, anagelsic dose, interviews differences in patients' and parents' perspectives in active vs non active departments, patient data	n=229	Patients responses or end points in care

Supplemental material 3, overview of full text articles n=33 (2 articles identified from reference lists)

Citation	Country	Title	Study design and Publication type	Data collected	sample size N=	Healthcare professionals	Recommendation of interprof education	Interprofessional education outcome Barr et al 2005 level	inclusion or exclusion (criteria)
Barnes et al 2014	UK	Physicians and nurses beliefs of phase 1 Trials in pedicatric oncology	Observational design	Survey	N=216	94 physicians and 122 nurses	y	n	exclusion No education intervention
Barr et al 2014	Central America	Asociación de Hemato-Oncología Pediátrica de Centro América (AHOPCA): a model for sustainable development in pediatric oncology	Review	N/A		nurses and postgraudate medical	n	n	exclusion Review
Bouri et al. 2017	Greece	The Impact of Pediatric Palliative Care Training on the Death Attitudes of Health Professionals	Pre/post intervention	Survey (validated)	N= 83	37 nurses, 21 psychologists, 10 pediatricians and 15 other disciplines (social workers, music therapists, physiotherapists)		2a	inclusion
Bradley-Eilertsen et al. 2009	Norway	Professional collaboration - support for children with cancer and their families - focus groups interview - a source of information and knowledge - professionals' perspectives	Research Support	Qualitative Focus groups	N=18	18 GP, pediatric oncologist, RN, teacher, educators for children with special needs, physiothearpist, SW, dietician, hospital minister, psychologist and public health nurse	y	n	exclusion No education intervention
Chen and Steingart 2011	USA	Cardiac disease and heart failure in cancer patients: is our training adequate to provide optimal care	Non systematic review	N/A		mono	n	n	exclusion No education intervention
Cheng 2015	USA	Do we need a formalized humanism and professionalism curriculum in pediatric hematology and oncology training	Letter to the editor	N/A		mono	n	n	exclusion No education intervention
Dalberg et al. 2013	USA	Pediatric Oncology Providers Perceptions of Barriers and Fac	Research Support Non-US Gov't	Qualitiative Focus groups (two validation focus groups)	N= 33	15 Physicians, 7 nurse practioners, two social workers, nine inpatient and outpatient nurses	y	n	exclusion No education intervention
de Freitas et al. 2014	Brazil	Degree of Knowledge of Health Care Professionals About Pain Management and Use of Opioids in Pediatrics	Observational design; Cross sectional study	Survey (not validated)	N=122 (93)	23 physicians, 2 pharmacists, 1 physiotherapist, 62 nursing technicians, 5 nursing assistants (=93) the study says 122 questionnaires?	y	n	exclusion No education intervention
Di Giulio et al. 2013	Inter European	Collaboration between doctors and nurses in children's cancer	Multicenter study Research Support Non-US Gov't	Projects implemented, publication of results, participants satisfaction	10 teams	10 teams of unspecified number of nurses and physicians		1, 2a & 2b	inclusion
Dobrasz et al. 2013	USA	Nurse-driven protocols for febrile pediatric oncology patients	Retrospective study	Medical records	All ED personel	Emergency nurses - all ED staff interdisciplinary quality improvement initiative - physician, nursing and pharmacy leaders decided to streamline AB to one product and pharmacy made it		3 & 4b	inclusion
Feraco et al. 2016	USA	Communication Skills Training in Pediatric Oncology: Moving Beyond Role Modelling	Review	Existing methods to enhance communication skills	not reported	n/a	y	n	exclusion Review
Fernandez et al 2006	Canada	Adolescents and Young Adults with cancer: An orphaned population	Review	N/A		mono		n	exclusion Review
Ferrari et al. 2010	Italy	Starting an adolescent and young adult program: some success stories and some obstacles to overcome	Review	N/A		pediatric and adult medical oncologists		n	exclusion Review
Finley et al. 2008	Canada	Action research: developing a pediatric cancer pain program in Jordan.	Research Support	Field observation, two focus group, unstructured interviews, individual intervies , audits of patient charts and pharmacy records	N=33	Four physicians, two nurses, one pharmasist , one "lead" researcher (visit one), additional staff; 14 nurses and 14 physicians		2a, 2b & 3	inclusion
Freyer et al. 2006	USA	In sickness and in health: transition of cancer-related care for	Review	N/A		n/a		n	exclusion No education intervention
Gibson et al. 2006	UK	Cancer-related fatigue in children and young people: Survey o	Qualitative research	Survey (not validated)	N=56	46 nurses, 10 doctors and other hps n=4	y	n	exclusion No education intervention
Greenfield & Hjorth 2013	UK	Late effects care as an emerging clinical specialty in paediatric oncology: how to prepare the workforce?	Review	Evidence for existing educational approaches	not reported	n/a	y	n	exclusion Review
Harris et al. 2004	USA	Palliative care in children with cancer: which child and when?	Research support, review	N/A		mono		n	exclusion No education intervention and monoprofessional
Henderson et al 2010	USA	Childhood cancer survivors: transition to adult-focused risk-based care	Review	N/A		Pediatric oncoogist and primary care	y	n	exclusion Review
Kusch et al. 2000	Germany	Structuring psychosocial care in pediatric oncology	Review	Theory (N/A)	not reported	n/a		n	exclusion No education intervention
Lamar et 2009	USA	Fertility preservation: state of the science and future research directions	Editorial	N/A		Occupational health and multidisciplinary medical staff	y	n	exclusion No education intervention and monoprofessional
Moody et al 2013	USA	Helping the helpers: mindfulness training for burnout in pediatric oncology—a pilot program.	Randomized controlled trial	Survey (validated)	N=48	Nurses,social workers, physicians, nurse practioners, psychologists, child-life specialists		2a & 3	inclusion
Neyrinck et al. 2015	Indonesia	Apheresis training for nurses and physicians around the world	Pre/post intervention	Survey (not validated)	N=70	38 apheresis nurses and 32 physicians		2b	inclusion
Patenaude et al. 2015	USA	Communication, Documentation, and Training Standards in Pediatric Psychosocial Oncology	Review	Consensus and evidences-based data on interprofessional communication, documentation, and training		n/a	y	n	exclusion review
Perilongo 2000	Italy	Guidelines for Integrated Activity between Pediatric Hematology-Oncology Nurses and Physicians	Guideline	N/A		Doctors and nurses	y	n	exclusion No education intervention
Sands et al. 2008	USA	Pediatric narrative oncology: interprofessional training to promote empathy, build teams and prevent burnout	Pre/post intervention and qualitative (Mixed Methods)	Surveys (validated) and focus group	N=19	6 physicians, 12 nurses, 1 psychosocial member		2a	inclusion
Solomon et al. 2005	USA	New and Lingering Controversies in Pediatric End-of-Life Care	Research Support	Survey (validated)	N=781 /796 ved egen regning	211 attending physicians, 116 house officers, 469/456 (?) nurses	y	n	exclusion No education intervention
Sung 2015	USA	Priorities for quality care in pediatric oncology supportive care	Perspective	N/A		n/a		n	exclusion No education intervention
Treadwell et al. 2002	UK	Using quality improvement strategies to enhance pediatric pain assessment	Research Support	Structured interviews adapted from the patient outcome questionnaire developed by American Pain society (validated)	N=T1:104, T2; 131	T1: 36 children and 68 staff (36 physicians, 29 nurses, and 3 psychosocial staff) , T2: 49 children and 82 staff (41 physicians, 35 nurses, six psychosocial staff)		2a, 3 & 4b	inclusion
Widger et al. 2016	Canada	Protocol: Evaluating the impact of a nation-wide train the trainer educational initiative to enhance the quality of palliative care for children with cancer	Research Support	Knowledge Transfer and Exchange Survey (validated?), interviews, Parent Survey (Validated)	N= + 600 endusers.	3-5 health professionals; training 45-80 trainers (+ 600 end users)		n	exclusion No education intervention
Wiener et al. 2015	USA	Threading the cloak: palliative care education for care providers of adolescents and young adults with cancer	Review	Epidemiologic, developmental, and psychosocial factors that make the provision of palliative care especially challenging i AYAs	not reported	n/a	y	n	exclusion Review
Yilmaz et al. 2010	Turkey	Health Professionals' Estimation of Cancer-Related Fatigue in Children	Descriptive	Survey (not validated)	N=56	44 nurses and 12 doctors	y	n	exclusion No education intervention
Zernikow et al. 2008	Germany	Stop the pain! A nation-wide quality improvement programme in paediatric oncology pain control	Comparative Study Evaluation Studies Multicenter Study Research Support	Survey on knowledge and attitudes (validated?), pain intensity ratings, anagelsic dose, interviews differences in patients' and parents' perspectives in active vs non active departments, patient data	N=229	76 heads of departments/supervising physicians, 46 ward physicians, 63 head nurses, 44 psychologists and social workers		2b & 4b	inclusion

Supplemental material 4, Languages other than English	
Main language	number of references
English	343
French	8
German	3
Polish	3
Spanish	3
Italian	2
Chinese	1
Dutch	1
Hrvatska (croatian)	1
Hungarin	1
Japanese	1
Portugese	1
Russian	1
srp (srpska) Bosnia-Hercegovina	1

Supplemental material 5, citations n# in Scopus, Journal and impact factor			
Citations	Citations in Scopus	Journal	impact factor
Bouri et al 2017(26)	0	International Journal of Caring Sciences	n/a
Dobrasz et al 2013	7	Journal of Emergency Nursing	0.795
Di Giulio et al 2013(31)	3	Europaen Journal of Oncology Nursing	1.826
Finley et al. 2008	26	Journal of Pain and Symptom Management	2.905
Moody et al 2013	34	Journal of Paediatric Oncology Nursing	0.987
Neyrinck et al 2015(27)	2	Journal of Clinical Apheresis	1.835
Sands et al. 2008(29)	35	Journal of Supportive Oncology	2.41
Treadwell et al 2002(28)	68	International Society for Quality in Health Care	2.342
Zernikow et al 2008(30)	21	European Journal of Pain	3.019
